# Critical steps in the path to using cessation pharmacotherapy following hospital-initiated tobacco treatment

**DOI:** 10.1186/s12913-019-4059-4

**Published:** 2019-04-24

**Authors:** Edward P. Liebmann, Taneisha S. Scheuermann, Babalola Faseru, Kimber P. Richter

**Affiliations:** 1 Department of Psychology, University of Kansas, Lawrence, KS USA; 2 Department of Preventive Medicine and Public Health, University of Kansas Medical Center, Mailstop 1008, 3901 Rainbow Blvd., Kansas City, KS 66160 USA

**Keywords:** Smoking cessation, Medication reconciliation, Tobacco use cessation devices, Patient discharge

## Abstract

**Background:**

Hospital-initiated smoking cessation interventions utilizing pharmacotherapy increase post-discharge quit rates. Use of smoking cessation medications following discharge may further increase quit rates. This study aims to identify individual, smoking-related and hospitalization-related predictors of engagement in three different steps in the smoking cessation pharmacotherapy utilization process: 1) receiving medications as inpatient, 2) being discharged with a prescription and 3) using medications at 1-month post-hospitalization, while accounting for associations between these steps.

**Methods:**

Study data come from a clinical trial (*N* = 1054) of hospitalized smokers interested in quitting who were randomized to recieve referral to a quitline via either warm handoff or fax. Variables were from the electronic health record, the state tobacco quitline, and participant self-report. Relationships among the predictors and the steps in cessation medication utilization were assessed using bivariate analyses and multivariable path analysis.

**Results:**

Twenty-eight percent of patients reported using medication at 1-month post-discharge. Receipt of smoking cessation medications while hospitalized (*OR* = 2.09, 95%CI [1.39, 3.15], *p* < .001) and discharge with a script (*OR* = 4.88, 95%CI [3.34, 7.13], *p* < .001) were independently associated with medication use at 1-month post-hospitalization. The path analysis also revealed that the likelihood of being discharged with a script was strongly influenced by receipt of medication as an inpatient (*OR* = 6.61, 95%CI [4.66, 9.38], *p* < .001). A number of other treatment- and individual-level factors were associated with medication use in the hospital, receipt of a script, and use post-discharge.

**Conclusions:**

To encourage post-discharge smoking cessation medication use, concerted effort should be made to engage smokers in tobacco treatment while in hospital. The individual and hospital-level factors associated with each step in the medication utilization process provide good potential targets for future implementation research to optimize treatment delivery and outcomes.

**Trial registration:**

Number: NCT01305928.

Date registered: February 24, 2011.

## Background

The rate of smoking among hospitalized patients (40%) exceeds that of the general population (13%) [1], suggesting that inpatient smokers are an important target for tobacco treatment intervention. Hospital-based treatment for tobacco dependence is effective [2]. Interventions that start in hospital and provide supportive contact for at least one-month post discharge increase quit rates by 37% [2]. Medications started during hospital stays are also effective for smoking cessation; a Cochrane review found that adding nicotine replacement therapy (NRT) to supportive inpatient intervention increased quit rates by 54% [2], and a recent clinical trial found that free NRT provided at discharge significantly increased quit rates independent of behavioral support [3]. Few hospitals, however, routinely provide or prescribe cessation medications to their patients. Freund et al. found that only 13% of hospital providers reported they offered or advised the use of cessation medications [4].

Hospital and patient-level factors influence patients’ pharmacotherapy use. Regan et al., 2012 found that smokers who received NRT during hospitalization were more likely to use it after discharge compared with those who did not use NRT in hospital [5]. Chui et al., 2018  found that predictors of NRT use among hospitalized smokers during their hospital stay and at discharge included heavy smoking and expressing interest in NRT for their next quit attempt [6]. Smokers who were admitted for a respiratory or cardiac problem were more likely to use a cessation medication at discharge [6]. Although these previous studies show that patient characteristics are associated with smoking cessation pharmacotherapy use, the process of how patients do or do not receive pharmacotherapy in hospital and prescriptions on discharge is unclear. Few studies have examined barriers to or facilitators of cessation pharmacotherapy administration in hospitals and use post-hospitalization [5–8]. Further, no studies have examined predictors of getting a prescription for cessation medication at hospital discharge.

Hospital systems of care could facilitate pharmacotherapy utilization. Medication reconciliation, a process that identifies patients’ current medications and is designed to improve quality and continuity of medication management [9], might influence the tobacco cessation pharmacotherapy process by carrying forward any current cessation prescriptions through the admission and discharge processes. Prior studies do not account for the causal relationships that the medication reconciliation process creates between receipt of medication in the hospital, receipt of a script on discharge, and utilization of medication post-discharge.

Insight into the factors that influence use of medications post-discharge may help hospital providers provide better care. The present study examines pathways and predictors of medication use at 1-month post-discharge. We used a multivariable path analysis to isolate the independent effects of a number of patient, hospitalization and smoking-related variables as well as the effects of factors in the medication reconciliation and prescribing process (such as using medications prior to admission, receiving medications as an inpatient, and receiving a script for medications on discharge).

## Methods

### Design and setting

This study is a secondary analysis of data from a clinical trial in which hospital inpatient smokers were randomized to either warm handoff or fax-referral for enrollment in state tobacco quitline services [10, 11]. The fax-referral arm was the usual tobacco treatment care provided by the hospital tobacco treatment staff. It consisted of a) assessing withdrawal; b) adjusting inpatient nicotine replacement to enhance patient comfort; c) arranging medication prescriptions on discharge; and d) developing a quit plan. Staff fax-referred patients to the quitline on the day they were discharged from the hospital. In the warm handoff arm, staff conducted usual care steps a-c, then immediately linked patients with the quitline via telephone for registration and completion of a quit plan with the quitline provider. Participants were enrolled in two Kansas hospitals with dedicated tobacco treatment interventionists on staff. Informed consent was obtained for all participants. The institutional review boards at both hospitals approved study protocols and measures.

### Participants and procedures

Eligible participants were planning to stay quit post-discharge, smoked within the past 30 days and were 18 years and older. Additional eligibility criteria and study procedures are reported elsewhere [10, 11]. Participants were identified through the electronic health record (EHR) and consented at bedside. Both study arms involved bedside tobacco treatment that included a smoking cessation booklet, discussing withdrawal symptoms, gauging interest in smoking cessation medications, and arranging for medication scripts both in the hospital and on discharge. No medications were provided as part of the study and prescribing hospital physicians were not part of the study team. In the warm handoff arm, study counselors called the quitline and transferred the call to the patients’ mobile or bedside hospital phone for enrollment and an initial counseling session; in fax-referral, counselors faxed an enrollment form to the quitline upon patient discharge.

### Measures

#### Patient-level measures

Variables from the baseline study survey included race, ethnicity and highest level of education. Patient birth date, sex and health insurance status were collected from the EHR. International Classification of Diseases-9 (ICD-9) codes were used for primary and secondary discharge diagnoses. Primary diagnoses were grouped into the major ICD-9 categories. We used a defined list of ICD-9 codes to identify patients with tobacco-related diseases [12]. Primary or secondary psychiatric disorders were indicated by ICD-9 codes 290–319, excluding 305.1 for tobacco use disorder.

#### Smoking-related measures

Variables related to patient smoking characteristics or tobacco treatment included the Heaviness of Smoking Index (HSI) [13], ,confidence in quitting/staying quit, and previous use of smoking cessation medications. Confidence in quitting was assessed by the item, “How confident are you that you will be able to quit/stay quit once you are discharged from the hospital?” The item was scored on a 1 (*Not at all confident*) to 5 (*very confident*) scale. Total quitline counseling calls was also collected for each participant and was provided by Optum, the state quitline contractor and a subcontractor on the project.

#### Hospitalization-related measures

Hospital length of stay in hours and whether or not the patient was admitted through the emergency department were collected from the EHR.

#### Smoking cessation pharmacotherapy utilization outcomes

We collected data on three potentially interrelated dependent variables related to cessation medications. Two were collected from the EHR: receipt of smoking cessation medication (NRT, bupropion, or varenicline) in-hospital and whether a prescription, or script, for smoking cessation medication was provided at discharge. The third variable—self-reported use of smoking cessation medication at 1-month post-discharge—was collected from participants via telephone survey.

### Statistical analysis

We included 984 of the original 1054 participants in this study who were reached for 1-month follow-up (93.4% of all participants). Path analysis was used to assess the conditional relationships of the independent variables and the three dependent medication-related variables. Path analysis uses a system of equations to test the viability of a multivariable theoretical or conceptual model and is akin to structural equation modeling with observed variables. Because path analysis is based upon a system of equations, it offers the ability to efficiently decompose covariation among a set of independent (i.e., exogenous) and dependent (i.e., endogenous). As result, path analysis can be used to test hypothesized direct and indirect effects [14].

**Fig. 1:**
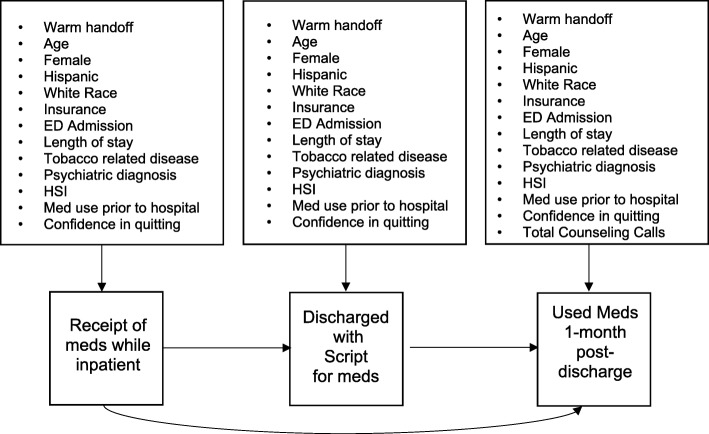
Schematic diagram for study path analysis The bulleted variables correspond to the independent variables in the model and the three medication-related variables correspond to the dependent variables. All arrows denote regression. In addition, each bulleted variable is a predictor of its respective outcome variable. *Note. ED = Emergency department, HSI = Heaviness of Smoking Index*

For the path analysis, the three dependent variables (receipt of pharmacotherapy while inpatient, discharge with a script for pharmacotherapy) were simultaneously regressed on a set of patient, smoking-related and hospitalization-related predictors. To account for interrelationships among the dependent variables, being discharged with a script was regressed on receipt of medication while inpatient and medication use at 1-month was regressed on discharge with a script for medication and receipt of medication while hospitalized. The path model is visually depicted in Fig. 1. The multivariable path analysis was estimated in Mplus, version 7.4 [15] using robust maximum likelihood (MLR) and Bayesian multiple imputation to account for missing data [16, 17].

## Results

### Relationships between medication outcomes

Twenty-eight percent (264/935) of the sample reported using medication at 1-month post-discharge. Twenty-four percent of participants received pharmacotherapy as an inpatient and 31% were discharged with a prescription. Of the participants who reported using pharmacotherapy at 1-month post-discharge, 60.5% were discharged with a prescription for medication and 43.6% received medication while hospitalized. The majority of patients discharged with a script for medication (51.5%) had received medication while hospitalized.

### Bivariate predictors of medication outcomes

Self-reported use of medication at 1-month was associated with older age, being in the fax-referral arm, shorter length of stay, tobacco related disease, nicotine dependence, previously using cessation medication and completing more counseling calls (Table 1). Being discharged with a script was also associated with dependence and previous medication use, but also with ED admission, psychiatric diagnosis and lower confidence in ability to quit. Lastly, using medication while inpatient was associated with a similar set of variables as the other outcomes, however with more robust effects of ED admission (Odds Ratio (*OR*) = 1.93, 95%CI [1.42, 2.66], *p* < .001) and psychiatric diagnosis (*OR* = 1.82, 95%CI [1.34, 2.45], *p* < .001). The statistically significant relationships shown in Table 1 suggest that using cessation medication while hospitalized, being discharged with a script and using medication at 1-month are influenced by a partially overlapping set of patient, hospitalization and smoking-realted factors, which we tested in a multivariable path analysis.

**Table 1:** Descriptive Statistics by Cessation Medication Use Outcome and Odds Ratios

	Received Meds Inpatient				Discharged with Script				Used Meds at 1-Month			
	24.4% (239/978)		95% CI		31.2% (305/979)		95% CI		28.2% (264/935)		95% CI	
		*OR*	LL	UL		*OR*	LL	UL		*OR*	LL	UL
Study arm, *N* (%)	115 (48.12)	0.86	0.64	1.15	152(49.84)	0.93	0.71	1.22	116 (43.94)	0.7	0.52	0.93*
Age (years), mean (SD)	49.71(13.19)	1	0.99	1.01	50.51 (12.34)	1.01	1	1.02	51.34 (12.13)	1.01	1	1.02*
Female, *N* (%)	141 (59)	1.21	0.9	1.62	182 (59.67)	1.28	0.98	1.69	157 (59.47)	1.22	0.92	1.64
Edu. > HS, *N* (%)	130 (54.39)	1.15	0.86	1.54	149 (48.85)	0.85	0.65	1.11	138 (52.27)	1.02	0.76	1.35
Hispanic, *N* (%)	13 (5.49)	0.85	0.44	1.56	18 (5.96)	0.95	0.53	1.65	13 (4.94)	0.8	0.4	1.47
White, *N* (%)	178 (74.48)	1.29	0.93	1.8	219 (71.8)	1.07	0.8	1.45	195 (73.86)	1.26	0.92	1.74
Insurance, *N* (%)												
Self-Pay	9 (3.77)	0.58	0.26	1.14	10 (3.28)	0.47	0.22	0.92*	15 (5.68)	1.03	0.54	1.88
Medicare	85 (35.56)	1.46	1.07	1.98*	103 (33.77)	1.34	1	1.79	82 (31.06)	1.09	0.8	1.48
Medicaid	78 (32.64)	0.89	0.65	1.21	113 (37.05)	1.17	0.88	1.56	96 (36.36)	1.06	0.79	1.42
Private/VA	67 (28.03)	0.87	0.63	1.2	79 (25.9)	0.73	0.54	0.99*	71 (26.89)	0.85	0.62	1.16
Admitted through ED, *N* (%)	170 (71.13)	1.93	1.42	2.66***	197 (64.59)	1.37	1.04	1.82*	168 (63.64)	1.26	0.94	1.7
Length of stay in hours^a^, median (IQR)	86.35 (81.75)	0.82	0.55	1.24	92.08 (87.7)	0.86	0.59	1.25	84.44 (92.06)	0.65	0.44	0.96*
Smoking related disease, *N* (%)	110 (46.03)	1.31	0.98	1.76	141 (46.23)	1.34	1.02	1.77*	124 (46.97)	1.4	1.05	1.87*
Psych. diagnosis, *N* (%)	104 (43.51)	1.82	1.34	2.45***	118 (38.69)	1.43	1.08	1.9*	78 (29.55)	0.82	0.6	1.11
HSI, mean (SD)	3.11 (1.53)	1.23	1.12	1.35***	3.12 (1.51)	1.26	1.16	1.37***	3.03 (1.59)	1.2	1.09	1.31***
Pre-admission Meds, *N* (%)	155 (64.85)	2.08	1.54	2.83***	196 (64.26)	2.16	1.64	2.86***	180 (68.18)	2.65	1.97	3.59***
Total counselling calls, median(IQR)	1(3)	1	0.92	1.09	1 (3)	1.04	0.96	1.13	2 (4)	1.16	1.07	1.26***
Confidence, mean (SD)	3.5 (1.13)	0.74	0.65	0.85***	3.67 (1.14)	0.87	0.77	0.98*	3.71 (1.07)	0.92	0.81	1.04
Inpatient Meds, *N* (%)	–	–	–	–	157 (51.48)	7.73	5.61	10.71***	115 (43.56)	3.66	2.67	5.02***
Discharged with script for Meds, *N* (%)	–	–	–	–	–	–	–	–	159 (60.46)	6.25	4.59	8.57***

*NRT* nicotine replacement therapy, *ED* emergency department, *IQR* interquartile range, *HS* high school, *OR* odds ratio, *HSI* Heaviness of Smoking Index, *SD* standard deviation, *VA* Veterans Administration, *CI* confidence interval, *LL* lower limit, *UL* upper limit, Meds = Smoking Cessation Medications (nicotine replacement, bupropion, or varenicline)

* p < .05; ** p < .01; *** p < .001

^a^Log(Length of stay) is used in bivariate analyses

### Path analysis

Table 2 presents the results from the multivariable path analysis. Being discharged with a script for medication (*OR* = 4.88, 95%CI [3.34, 7.13], *p* < .001) and using medication while inpatient (*OR* = 2.09, 95%CI [1.39, 3.15], *p* < .001) were independently associated with using medication at 1-month. Using cessation medication as an inpatient was robustly associated with being discharged with a script (*OR* = 6.61, 95%CI [4.66, 9.38], *p* < .001).

**Table 2:** Model Results from Multivariable Path Analysis of Steps in Cessation Pharmacotherapy Engagement

	Received Meds Inpatient			Discharged with Script			Used Meds at 1-Month		
	*OR*	95% CI		*OR*	95% CI		*OR*	95% CI	
Predictor		LL	UL		LL	UL		LL	UL
Warm handoff	0.92	0.67	1.25	1.02	0.75	1.38	0.57	0.41	0.79**
Age, years	0.99	0.97	1	1	0.99	1.01	1.01	0.99	1.02
Female	1	0.73	1.37	1.06	0.77	1.46	0.92	0.65	1.30
Hispanic	1.12	0.56	2.22	1.26	0.69	2.32	1.01	0.47	2.15
White race	1	0.68	1.46	0.77	0.54	1.10	1.20	0.80	1.80
Insurance									
None	0.51	0.23	1.14	0.60	0.26	1.39	1.94	0.94	4.01
Medicare	1.46	0.96	2.20	1.23	0.81	1.87	0.86	0.55	1.36
Medicaid	0.91	0.61	1.37	1.45	0.98	2.15	1.22	0.81	1.85
Admitted through ED	2.12	1.52	2.95***	1.11	0.80	1.52	1.08	0.76	1.52
log(LOS^a^)	1.01	0.67	1.54	0.85	0.56	1.31	0.56	0.34	0.90*
Smoking related disease	1.27	0.91	1.78	1.21	0.87	1.68	1.04	0.73	1.48
Psychiatric diagnosis	1.73	1.24	1.78**	1.17	0.83	1.64	0.57	0.39	0.83**
HSI	1.21	1.09	1.34***	1.21	1.10	1.33***	1.06	0.95	1.19
Used meds prior to admission	2.02	1.45	2.81***	1.82	1.30	2.55***	2.00	1.39	2.88***
Confidence	0.80	0.70	0.92**	1.04	0.90	1.20	1	0.86	1.13
Used meds in hospital	–	–	–	6.61	4.66	9.38***	2.09	1.39	3.15***
Discharged with script for meds	–	–	–	–	–	–	4.88	3.34	7.13***
Total counselling calls	–	–	–	–	–	–	1.22	1.04	1.35***

*NRT* nicotine replacement therapy, *ED* emergency department, *LOS* length of stay, *HSI* Heaviness of Smoking Index, *OR* odds ratio, *UL* Upper Limit, *LL* lower limit, Meds = Smoking Cessation Medications (nicotine replacement, bupropion, or varenicline)

* p < .05; ** p < .01; *** p < .001

^a^Length of stay is in hours

Use of medication while inpatient was associated with admission through the ED, having a psychiatric diagnosis, nicotine dependence, past smoking cessation medication use and lower confidence in ability to quit. After controlling for medication use while inpatient, nicotine dependence and past medication use were the only factors with significant odds of being discharged with a script for medication. After controlling for the effects of a) receiving inpatient pharmacotherapy and b) being discharged with a script for medication, the likelihood of medication use at 1-month was associated with a shorter length of stay, not having a psychiatric diagnosis, using medication prior to hospitalization and engagement in a greater number of counselling sessions post-hospitalization.

## Discussion

Our findings demonstrate that receiving smoking cessation medication as an inpatient and being discharged with a script for medication are each independently associated with patients’ utilization of pharmocotherapy at 1-month post-hospitalization. These effects were robust after accounting for both individual and treatment related factors.

The present study extends the literature by accounting for the causal pathway of medication provision. Specifically, we examined discharge with a script for medication as both a dependent variable and a predictor of cessation medication use post-discharge. The likelihood of being discharged with a script was strongly influenced by receipt of medication as an inpatient and was robustly associated with medication use at follow-up. The strength of this association suggests a high degree of continuity between leaving the hospital with a script and ultimately using medication post-discharge. Implementation research on how to increase the rate of provision of scripts for cessation medication during discharge and how to increase the likelihood that these scripts are filled may lead to greater medication utilization, and in turn, improved quit rates among recently hospitalized smokers.

Hospital-initiated smoking cessation interventions are most effective when they extend 1-month post-discharge and their efficacy is enhanced with the addition of pharmacotherapy [2]. The multivariable model showed that cessation medication use at 1-month was associated with multiple factors above and beyond the medication pathway described above. Notably, medication use was associated with greater engagement in counseling and not having a psychiatric diagnosis. These results suggest that counseling that is initiated in the hospital and continued via quitline post-discharge can reinforce the use of cessation medication, which is consistent with previous research [5, 18]. In addition, smokers with psychiatric diagnoses, or psychosis [18], may need more intensive support in order to facilitate treatment utilization post-hospitalization.

Findings from this study confirm those of previous studies. Previous research has found that smoking cessation medication use prior to hospitalization [6, 8], nicotine dependence [6–8] and less confidence in ability to not smoke [8] are associated with medication use while hospitalized. Consistent with the present results, post-discharge medication use has been associated with inpatient medication use [5, 18], medication use prior to hospitalization [5], shorter length of stay [5] and tobacco related disease [6]. These results lend support to findings from the general population in which smokers who elected to use NRT were more nicotine dependent, less confident in their ability to quit and generally possessed more risk factors for cessation failure [19]. Similar to findings from the main outcome study [10], patients receiving the warm handoff intervention were less likely to use medications post discharge-likely because they had shorter visits with inpatient counselors who may have, compared to quitline counselors, put more stress on the importance of using medications post-discharge.

In addition, this study provides additional information on elements of the hospitalization context that may influence the likelihood of using smoking cessation medication post-discharge. In addition to length of stay, admission through the ED increased the likelihood of receiving medication while hospitalized and of being discharged with medication, although the effect on discharge did not remain significant in the multivariable analysis. One possible explanation for the effect of ED admission on receipt of medication is that waiting to be seen in the ED may function as a period of forced abstinence, thus patients may be in withdrawal and be more likely to request relief and/or be offered cessation medication once admitted. Other possible explanations include: ED patients have higher smoking prevalence than other hospital patients [20], they often present with tobacco-related illnesses or need surgical procedures that will require smoking abstinence for optimal wound healing [21], and they are more likely to receive smoking cessation treatment than non-ED patients [22]. Future research is needed to identify other aspects of the hospitalization context that influence treatment engagement.

### Limitations

The present study had two major limitations. First, the reliance upon self-report for medication use at 1-month raises the possibility of participant misreporting. In addition, because intending to stay quit post-discharge was an inclusion criterion for the study, the presented results may not be generalizable to inpatient smokers who are not prepared to make a quit attempt. The study was conducted in two hospitals in the U.S., which may not be representative of community hospitals or hospitals in other countries. In addition, hospitals that do not have dedicated tobacco interventionists might achieve different pharmacotherapy utilization results. For example, if advanced practice providers (APPs) such as nurse practitioners took on the task of treating tobacco dependence, inpatient utilization, discharge prescription, and hence outpatient utilization rates could be higher because APPs have the ability to write prescriptions for medications. The present analysis also had several strengths, including the relatively large sample size, the use of variables from multiple sources including the EHR, the quitline provider, and study participants; and the use of multivariable path analyses that controlled for hospital procedures—such as medication reconciliation at admission and discharge—that increase the likelihood that past medication receipt predicts future medication receipt.

## Conclusions

In sum, smoking cessation medication post-discharge is independently associated with receiving medication while inpatient and being discharged with a script for medication, even after accounting for other hospital treatment and individual characteristics. Continued efforts to engage hospitalized smokers in treatment while inpatient and in transition from the hospital are necessary to promote sustained medication use post-hospitalization. Hospitalized smokers intending to stay quit may be highly vulnerable to relapse post-discharge without sustained medication use.

## Abbreviations

ED: Emergency Department
EHR: Electronic health record
HSI: Heaviness of Smoking Index
ICD-9: International Classification of Diseases-9
LOS: Length of stay
MLR: Robust maximum likelihood
NRT: Nicotine replacement therapy
OR: Odds ratio
